# Capability of well-being: validation of the Hungarian version of the ICECAP-A and ICECAP-O questionnaires and population normative data

**DOI:** 10.1007/s11136-020-02542-1

**Published:** 2020-05-28

**Authors:** Petra Baji, Miklós Farkas, Ágota Dobos, Zsombor Zrubka, László Gulácsi, Valentin Brodszky, Fanni Rencz, Márta Péntek

**Affiliations:** 1 Department of Health Economics, Corvinus University of Budapest, 8 Fővám tér, Budapest, 1093 Hungary; 2 Department of Accounting and Finance, University of Bristol, Bristol, UK; 3 Corvinus Center for Foreign Language Education and Research, Corvinus University of Budapest, Budapest, Hungary; 4 Hungarian Academy of Sciences, Premium Postdoctoral Research Programme, Budapest, Hungary

**Keywords:** Capability, ICECAP-A, ICECAP-O, Validation, Psychometrics, Reliability

## Abstract

**Purpose:**

We aimed to develop and assess the psychometric characteristics of the Hungarian language version of two well-being capability measures, the ICEpop CAPability measure for Adults/Older people (ICECAP-A/-O), and to establish population norms.

**Methods:**

A cross-sectional survey was performed involving a representative sample of the Hungarian population. Socio-demographic characteristics, the use and provision of informal care were recorded. The Minimum European Health Module (MEHM), EQ-5D-5L, WHO-5 well-being index, happiness and life satisfaction visual analogue scale (VAS), Satisfaction with Life Scale (SWLS) measures were applied alongside the ICECAP-A (age-group 18–64) and ICECAP-O (age-group 65+).

**Results:**

Altogether 1568 and 453 individuals completed the ICECAP-A/-O questionnaires, respectively. Cronbach’s alpha was 0.86 for both measures (internal consistency). Subgroup analyses showed positive associations between ICECAP-A/-O scores and marital status, employment, income, health status (MEHM) and informal care use (construct validity). Pearson correlations were strong (*r* > 0.5; *p* < 0.01) between ICECAP-A/-O indexes and EQ-5D-5L, WHO-5, happiness and satisfaction VAS and SWLS scores (convergent validity). The age, education, and marital status were no longer significant in the multiple regression analysis. Test–retest average (SD) scores were 0.88 (0.11) and 0.89 (0.10) for the ICECAP-A, and equally 0.86 (0.09) for the ICECAP-O (reliability).

**Conclusion:**

This is the first study to provide ICECAP-A/-O population norms. Also, it is the first to explore associations with WHO-5 well-being index which, alongside the MEHM measures, enable estimates from routinely collected international health statistics. The Hungarian ICECAP-A/-O proved to be valid and reliable measurement tools. Socio-demographic characteristics had minor or no impact on ICECAP-A/-O. Other influencing factors deserve further investigation in future research.

**Electronic supplementary material:**

The online version of this article (10.1007/s11136-020-02542-1) contains supplementary material, which is available to authorized users.

## Introduction

Ageing of the populations and the increasing prevalence of chronic diseases in Europe poses significant burden on the health and social care systems [1]. The economic evaluation of informal and social care interventions, as well as their contribution to societal welfare, have received increased attention in the past years [2, 3]. In current health economic analyses and guidelines, the health-related quality of life (HRQoL) outcomes are used and recommended as golden standard. However, there is a growing interest in new emerging measures, which capture wider concept of well-being [4].

ICECpop CAPability (ICECAP) measures have been developed to be used in economic evaluations to capture aspects of well-being beyond health and HRQoL. The ICECAP measures are based on Amartya Sen's capability approach which defines well-being in terms of an individual's ability and capability to ‘do’ certain things that are important in life [5]. The ICECAP-A instrument has been developed for use among the general adult population (18 years and older) [6], while the ICECAP-O instrument addresses important aspects of life of the older population (65 years and over) [7]. Helter et al. identified 14 capability instruments, but only the ICECAP-A, ICECAP-O and the Adult Social Care Outcomes Toolkit (ASCOT) instruments reported both psychometric properties and valuation studies reflecting the preferences (utility) of the general public [8]. The use of ICECAP instruments is evolving in economic evaluations. A recent systematic review by Proud et al. identified 22 studies where the ICECAP-O measure was applied in economic evaluations [9]. On the regulatory level, the National Institute for Health and Care Excellence (NICE) recommends the use of ICECAP-O for measuring the impact of social care interventions, while in the Netherlands, ICECAP-O is recommended to be used for evaluations of long-term conditions [10, 11].

Several studies assessed the validity of the ICECAP measures [8, 9], however most of the literature focuses on the UK, where the measures were originally developed [12–14], and on other English speaking countries, such as Australia [15, 16] and Canada [17, 18]. Other language versions of the ICECAP-O instrument have been developed and validated in Dutch [19], German [20], Spanish [21] and Swedish [22]. ICECAP-A has been validated in Chinese [23] and in German [11]. Nevertheless, no language versions have been available so far for the Central and Eastern European countries. Moreover, all ICECAP-A/-O studies (except an ICECAP-O study in Australia [16]) involved either specific samples (e.g. patient groups or informal caregivers), or could achieve only partial representativeness in population-based samples due to online recruitment, relatively small sample size or higher response rates in specific groups [13, 14, 24]. In European countries no population norms have been established that are based on a large sample, and can be considered representative along key demographic characteristics.

The primary aim of our study was, therefore, to develop the Hungarian versions of the ICECAP-A and ICECAP-O instruments, assess their construct validity, and test–retest their reliability among the Hungarian general population. Secondarily, we aimed to establish population normative data with both measures.

## Methods

### The survey

We designed and conducted a large population health survey in Hungary (year 2019) and developed the Hungarian version of the ICECAP-A and -O as part of this research. Computer-assisted personal interviews were conducted among the Hungarian adult general population (*N* = 2023). The recruitment of the respondents and the interviews were carried out by a subcontractor (New Land Media Kft., Survey Company). Quotas were applied based on the number and composition of the population (National Central Statistical Office) to obtain a representative sample in terms of age, gender and residence [25]. Ethical approval was obtained from the Hungarian Medical Research Council (no. 10058-3/2019/EKU). Respondents were informed that the participation in the survey was voluntary, the data would remain anonymous, impersonal and would be used solely for scientific purposes. Respondents needed to give their informed consent before the start of the survey.

In this paper our focus is on the ICECAP-A/-O results, but the survey covered five major modules: (1) socio-demographics (such as age, gender, education, marital status, employment status, household size, monthly net household income, place of residence) (2) health (3) well-being, happiness and satisfaction with life (4) major life events in the last 12 months and (5) experience with informal care either as a current caregiver (for the last 2 weeks) or recipient (in the past 3 months). A detailed definition of informal care was provided for the respondents. In brief, informal care was determined as a non-paid support or care, provided for family members or acquaintances in need of help due to health problems or ageing. The applied measurement tools introduced in the next sections were presented in the same order to each respondent. For the ICECAP-A, ICECAP-O and EQ-5D-5L questionnaires, we used self-completed paper based versions, and the data were recorded into the electronic database.

### The instruments

#### The ICECAP-A and ICECAP-O and the development of their Hungarian versions

The ICECAP-A (for age group 18+) and ICECAP-O (for age group 65+) are preference-based measures of well-being capabilities, developed for use in economic evaluations [6, 7, 12]. Both measures cover five domains of well-being. The ICECAP-A items are (1) Attachment (an ability to have love, friendship and support); (2) Stability (an ability to feel settled and secure); (3) Achievement (an ability to achieve and progress in life); (4) Enjoyment (an ability to experience enjoyment and pleasure) and (5) Autonomy (an ability to be independent). The ICECAP-O items are the following: (1) Attachment (love and friendship); (2) Security (thinking about the future without concern); (3) Role (doing things that make you feel valued); (4) Enjoyment (enjoyment and pleasure); (5) Control (independence). A 4-level response scale is applied for each item and respondents are asked to indicate the one that best describes their overall quality of life at the moment. Scores range from 0, which represents ‘no capability’ to 1, which represents ‘full capability’ based on the tariff sets for the instruments developed using best–worst scaling methods [26, 27]. Tariffs are currently available only for the UK population, so we used those to calculate index scores. In this study, ICECAP-A scores were calculated for respondents below the age of 65 (< 65) and ICECAP-O scores for respondents (65+).

The Hungarian language versions of the ICECAP-A and ICECAP-O questionnaires were developed in accordance with available guidance on the topic [28]. In brief, independent forward- and back-translations were undertaken. Differences were discussed among the investigators and they were reviewed by the copyright owner of the original English ICECAP measures. With the final versions, semi-structured interviews (*N* = 10) were conducted with the aim to assess the comprehensiveness and the relevance of the content.

#### Minimum European Health Module (MEHM)

The MEHM health status measure consists of three general questions characterizing three different concepts of health: (1) self-perceived health in general (very good/good/fair/bad/very bad); (2) long-standing illness (3) long-standing activity limitations due to health problems measured via the Global Activity Limitation Indicator (GALI) (severely limited/limited but not severely/not limited at all) [29].

#### The EQ-5D-5L

The EQ-5D-5L is a generic health status measure which distinguishes between five health domains, i.e. Mobility, Self-care, Usual activities, Pain/discomfort, Anxiety/depression [30]. Respondents are asked to indicate the problem level (1—no, 2—slight, 3—moderate, 4—severe and 5—unable/extreme problems) that best describes their health ‘today’. Due to the lack of country-specific value set for Hungary [31], we used the tariffs for England in our study (value range: − 0.285 to 1) to calculate EQ-5D-5L index score [32]. In the second part of the questionnaire respondents are asked to value their health that day on a EQ VAS, a vertical 0–100 visual analogue scale (0—worst, 100—best health state the respondent can imagine).

#### World Health Organization-Five Well-Being Index (WHO-5)

The WHO-5 is a short self-reported measure of mental well-being [33, 34]. It consists of five statements, which respondents rate in relation to the past 2 weeks on a 6-point Likert-scale (0—“at no time”; 5—“all of the time”) answer. The WHO-5 index is calculated by summing up the scores which is multiplied by 4 to give the final score between 0 and 100.

#### Happiness and satisfaction with life visual analogue scales (VAS)

We used 0–10 VAS to measure respondents’ current happiness and satisfaction with life (0—completely unhappy/not satisfied at all; 10—completely happy/completely satisfied).

#### The Satisfaction with Life Scale (SWLS)

The SWLS is a five-item instrument, designed to measure global cognitive judgments of satisfaction with one's life [35, 36]. Respondents are asked to indicate their agreement with each of the five statements on a seven-point Likert-scale (1—“Strongly disagree”, 7—“Strongly agree”). The index is the summary of the scores, ranging from 5 to 35 with a score of 20 representing a neutral point on the scale (5–9: extremely dissatisfied; 31–35: extremely satisfied.)

### Statistical analysis

The psychometric properties of the ICECAP-A and ICECAP-O instruments were evaluated in relation to socio-demographic characteristics, health status, major life events and health-related quality of life (EQ-5D-5L) as well as well-being measures (WHO-5, happiness and satisfaction VAS, SWLS).

We used the terminology from the COnsensus-based Standards for the selection of health status Measurement INstruments (COSMIN) taxonomy to describe validity while investigating psychometric properties of the instrument [37]. We investigated internal consistency, construct validity (hypothesis testing, including convergent validity) and reliability (and test–retest reliability).

To assess the internal consistency, Cronbach’s alphas were calculated [38]. Alpha ranges from 0 to 1 (0.7–0.8: acceptable, 0.8–0.9: good > 0.9: excellent internal consistency) [39].

Construct validity was assessed via one-way subgroup comparisons and by multiple regression analysis. We investigated whether the ICECAP-A/ICECAP-O scores can differentiate between groups hypothesized to differ in their levels of the construct capability-well-being, i.e. among different socio-demographic characteristics, health status and major life events. Subgroup comparisons were carried out by one-way ANOVA tests, associations were also explored by OLS multiple regression analysis. Based on previous literature [9, 11, 12, 14], we expected positive association with the following variables: being married/living with a partner, having higher income, being employed/having a paid job, living with others. A positive relationship was also expected between ICECAP-A/ICECAP-O scores and better health. On the other hand, we expected no association with gender, and no or negative association with age. We also investigated if capabilities differed by informal caregiving situation. Coast et al. found positive but not significant relationship [12, 13].

To assess convergent validity, Pearson’s correlation coefficients were calculated between ICECAP-A/ ICECAP-O scores and related measures of health-related quality of life and well-being (EQ-5D-5L index, EQ VAS, WHO-5, happiness and satisfaction VAS, SWLS) and Spearman’s rho correlations between domains of these measures. Correlations were considered strong if the coefficient was over 0.5, moderate between 0.3 and 0.5 and weak under 0.3 [40]. Based on previous literature we expected moderate / strong correlation with the EQ-5D-5L and SWLS measures [11]. Positive association between ICECAP scores and WHO-5 scores were also assumed.

To assess the test–retest reliability of the ICECAP-O and ICECAP-A scores, 5% of the respondents were asked to participate in a follow up measurement right after the interview. For each questionnaire item, we calculated the percentage of agreement on each item. We calculated intra-class correlation coefficients (ICC), using a two-way mixed model of absolute agreement. The ICC can range from 0.00 (no stability/agreement) to 1.00 (perfect agreement). Based on Koo et al. [41], agreement is considered poor for ICC values below 0.5, moderate between 0.50 and 0.749, good between 0.750 and 0.900 and excellent above 0.90.

In all types of analysis, a 5% significance level was applied. All analyses were undertaken using Stata version 13.

## Results

### The sample

The sample size was 2023 respondents (50.1% women) with the average age of 48.7 years (SD = 17.9). All but two respondents answered all questions on the ICECAP-A instrument (*n* = 1568), and all respondents aged 65+ answered all questions on the ICECAP-O (*n* = 453). Socio-demographic characteristics, health status (MEHM) and informal care experience of the sample are presented in Tables 1 and 2, respectively. Summary statistics for the standard health status and well-being measures are provided in Table 3.

**Table 1:** Sample characteristics and population norms for the ICECAP-A and ICACAP-O scores by socio-demographic groups of the sample

Variables	Age-group 18–64 years			Age-group 65 years and over		
	*N*	%	ICECAP-A score Mean (SD)	*N*	%	ICECAP-O score Mean (SD)
Total	1568		0.89 (0.13)	453		0.83 (0.15)
Gender			*p* = 0.4673			*p* = 0.8391
Women	787	50.2	0.89 (0.13)	226	49.9	0.83 (0.14)
Men	781	49.8	0.90 (0.13)	227	50.1	0.83 (0.16)
Age category (years)			*p* = 0.0000			*p* = 0.0000
18–24	208	13.3	0.92 (0.10)			
25–34	308	19.6	0.92 (0.10)			
35–44	386	24.6	0.89 (0.13)			
45–54	332	21.2	0.91 (0.11)			
55–64	334	21.3	0.84 (0.15)			
65–74				267	58.9	0.85 (0.13)
75–84				145	32.0	0.81 (0.16)
85+				41	9.1	0.72 (0.18)
Education^a^			*p* = 0.0000			*p* = 0.0025
Primary	576	36.7	0.87 (0.15)	268	59.2	0.81 (0.16)
Secondary	662	42.2	0.91 (0.11)	106	23.4	0.86 (0.11)
Tertiary	330	21.0	0.92 (0.09)	79	17.4	0.86 (0.15)
Employment^b^			*p* = 0.0000			*p* = 0.0682
Employed full time/self-employed	1197	52.8	0.92 (0.10)	14	3.1	0.89 (0.14)
Working part time	42	1.9	0.80 (0.18)	4	0.9	0.92 (0.06)
Pensioner	84	3.7	0.84 (0.14)	429	94.7	0.83 (0.15)
Disability pensioner	48	2.1	0.66 (0.21)	6	1.3	0.73 (0.19)
Student	776	34.2	0.92 (0.10)			
Unemployed (seeking for a job)	53	2.3	0.79 (0.18)			
Unemployed (not seeking for a job)	12	0.5	0.82 (0.13)			
Housewife/husband	25	1.1	0.93 (0.12)			
Other	31	1.4	0.87 (0.17)			
Having a paid job			*p* = 0.0000			*p* = 0.0151
No	312	19.9	0.83 (0.17)	422	93.2	0.82 (0.15)
Yes	1256	80.1	0.91 (0.11)	31	6.8	0.89 (0.12)
Settlement type			*p* = 0.4265			*p* = 0.9968
Budapest	309	19.7	0.90 (0.09)	90	19.9	0.83 (0.11)
Other town	811	51.7	0.89 (0.13)	249	55.0	0.83 (0.16)
Village	448	28.6	0.89 (0.13)	114	25.2	0.83 (0.16)
Marital status			*p* = 0.0000			*p* = 0.0000
Married	714	45.5	0.90 (0.11)	224	49.4	0.86 (0.11)
Partnership	292	18.6	0.91 (0.13)	8	1.8	0.86 (0.14)
Single	378	24.1	0.89 (0.14)	9	2.0	0.77 (0.31)
Widow/widower	42	2.7	0.80 (0.19)	165	36.4	0.80 (0.17)
Divorced	139	8.9	0.86 (0.15)	46	10.2	0.80 (0.17)
Other	3	0.2	0.90 (0.01)	1	0.2	0.27 (0.00)
Married/partnership			*p* = 0.0000			*p* = 0.0000
No	562	35.8	0.88 (0.15)	221	48.8	0.80 (0.18)
Yes	1006	64.2	0.90 (0.11)	232	51.2	0.86 (0.11)
Living with someone in the household			*p* = 0.0003			*p* = 0.0333
No	201	12.8	0.86 (0.16)	170	37.5	0.81 (0.17)
Yes	1367	87.2	0.90 (0.12)	283	62.5	0.84 (0.13)
Per capita net income category (quintiles)			*p* = 0.0000			*p* = 0.0012
1st quintile	211	20.5	0.82 (0.18)	60	19.2	0.76 (0.20)
2nd quintile	250	24.2	0.89 (0.12)	77	24.7	0.85 (0.11)
3rd quintile	206	20.0	0.90 (0.11)	106	34.0	0.82 (0.16)
4th quintile	183	17.7	0.91 (0.10)	49	15.7	0.87 (0.10)
5th quintile	181	17.6	0.93 (0.09)	20	6.4	0.87 (0.10)

ANOVA tests were applied

^a^Primary: no matriculation in high school; secondary: high school with matriculation; tertiary: professional training with diploma in higher education, BSc, MSc or PhD diploma

^b^In case of multiple employments (e.g. student and employed), respondents were asked to indicate the employment status that was the most characteristic for them

**Table 2:** ICECAP-A (age: < 65) and ICECAP-O (age: 65 and over) scores by health status

Variables	Age-group 18–64 years			Age-group 65 years and over		
	*N*	%	ICECAP-A score Mean (SD)	*N*	%	ICECAP-O Mean (SD)
Self-reported health			*p* = 0.0000			*p* = 0.0000
Poor	24	1.5	0.63 (0.2)	44	9.7	0.65 (0.2)
Fair	155	9.9	0.74 (0.18)	207	45.7	0.8 (0.14)
Good	585	37.3	0.89 (0.11)	166	36.6	0.88 (0.11)
Very good	461	29.4	0.93 (0.08)	28	6.2	0.94 (0.07)
Excellent	343	21.9	0.95 (0.09)	8	1.8	0.97 (0.04)
Long standing illness			*p* = 0.0000			*p* = 0.0000
No	1235	78.9	0.92 (0.1)	145	32.3	0.9 (0.09)
Yes	330	21.1	0.8 (0.17)	304	67.7	0.8 (0.16)
Global activity limitation indicator (GALI)			*p* = 0.0000			*p* = 0.0000
Severly limited	21	1.3	0.63 (0.21)	44	9.7	0.64 (0.22)
Limited, but not severly	153	9.8	0.74 (0.18)	176	38.9	0.79 (0.14)
Not limited	1393	88.9	0.92 (0.10)	232	51.3	0.89 (0.09)
Informal caregiver			*p* = 0.6068			*p* = 0.8409
Yes	66	4.2	0.90 (0.13)	15	3.3	0.82 (0.14)
No	1502	95.8	0.89 (0.12)	438	96.7	0.83 (0.15)
Informal care recipient			*p* = 0.0000			*p* = 0.0000
No	1496	95.9	0.9 (0.12)	343	76.2	0.86 (0.13)
No, but would need	7	0.4	0.69 (0.23)	6	1.3	0.69 (0.22)
Yes	57	3.7	0.76 (0.17)	101	22.4	0.74 (0.17)

ANOVA tests were applied

**Table 3:** Sample summary statistics for age, EQ-5D-5L, EQ VAS, ICECAP-A, ICECAP-O, happiness and satisfaction

	Total			Age-group 18–64 years			Age-group 65 years and over		
	*N*	Mean	SD	*N*	Mean	SD	*N*	Mean	SD
Age, years	2023	48.72	17.88	1570	41.62	13.15	453	73.32	7.01
ICECAP-A (0–1)	1568	0.89	0.13	1568	0.89	0.13	NA	NA	NA
ICECAP-O (0–1)	453	0.83	0.15	NA	NA	NA	453	0.83	0.15
EQ-5D-5L^a^ (− 0.285 to 1)	2020	0.92	0.15	1567	0.95	0.12	453	0.80	0.20
EQ VAS (0–100)	2023	81.59	17.42	1570	85.74	14.92	453	67.21	17.83
Happiness (0–10)	2021	7.64	1.95	1568	7.91	1.81	453	6.71	2.13
Satisfaction (0–10)	2022	7.53	2.01	1570	7.76	1.90	452	6.75	2.16
WHO-5 (0–100)	2023	71.98	18.02	1570	74.29	16.79	453	63.98	19.78
SWLS (0–35)	2023	24.56	6.19	1570	24.65	6.18	453	24.26	6.24

*NA* not applicable

^a^Calculated with value set for England [32]

### Population norms for the ICECAP-A and ICECAP-O

**Fig. 1:**
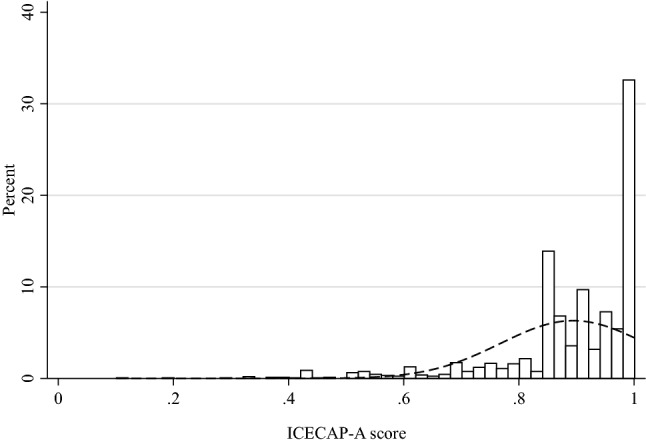
Distribution of ICECAP-A (age-group 18–64) scores

**Fig. 2:**
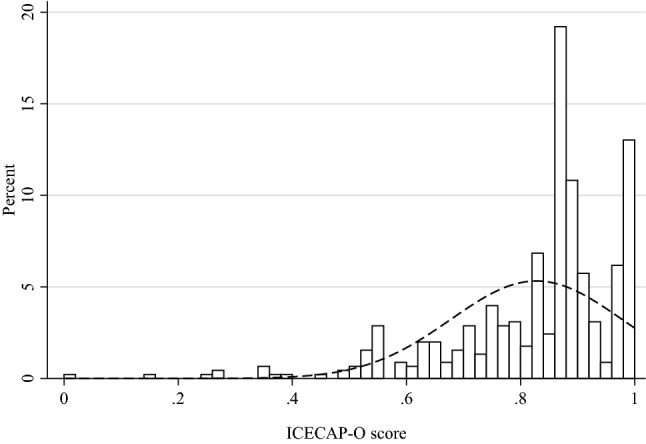
Distribution of ICECAP-O (age-group 65+) scores

The mean ICECAP-A and ICECAP-O scores were 0.89 (SD = 0.13) and 0.83 (SD = 0.15), and the distribution of scores are presented in Figs. 1 and 2, respectively. Distribution of answers on the ICECAP-A and ICECAP-O items are presented in Online Resource 1. The modal response of the ICECAP-A was the top level (level 4) of capability across all domains but Achievement, where the most common answer was the second best level (level 3: 44.6%), followed by the top level (level 4: 43.4%). For the ICECAP-O, the modal answer was the second best level (level 3) across each of the five domains.

Average ICECAP-A and -O scores of subgroups by socio-demographic characteristics are provided in Table 1. In the one-way ANOVA analysis, significant differences were found in both ICECAP-A and ICECAP-O scores by age category, education level, employment status (only ICECAP-A), marital status, if the respondent was living with someone, income quintiles (Table 1) and by all health indicators (Table 2). Furthermore, ICECAP scores were significantly lower if the respondent was an informal care recipient. No significant differences were observed by gender, settlement type, or if the respondent was an informal caregiver on either of the two instruments. Nonetheless, age, education and marital status variables were no longer significant in the multiple regression analysis (Online Resource 2). Also, employment (having a paid job) and income level were significant determinants only for the ICECAP-A instrument. Furthermore, regression results show that respondents living in the capital had significantly lower ICECAP-A scores.

### Construct validity and internal consistency

The differences found between subgroups as detailed in the section above support the good construct validity of the instruments. In addition, both ICECAP-A and ICECAP-O scores were significantly lower if some of the major life events happened to the respondents in the last 12 months (Online Resource 3), i.e. Death of a close relative or friend; Serious financial worries or debts; Problems with parents or close relatives; Other serious illness to the respondent. ICECAP-A scores were also lower if the wage earner in the household lost their job; in the case of problems at work and in the case of a serious accident or injury to the respondent. While ICECAP-O scores were significantly lower if problems were reported with children.

Cronbach’s alpha was 0.863 for the ICECAP-A and 0.864 for the ICECAP-O instrument, indicating good internal consistency.

### Convergent validity

#### Correlation between indexes

Correlation coefficients between the ICECAP-A/-O scores, domains and the other standard measures are presented in Table 4. We observed strong correlation of the ICECAP measures with EQ-5D-5L index, EQ VAS, WHO-5 score, happiness and satisfaction with life VAS; moderate/strong correlation with SWLS score. Four ICECAP-A items had moderate correlations with all the indexes, while Autonomy had only weak to moderate correlation. Correlation coefficients were slightly higher for the ICECAP-O instrument (Table 4).

**Table 4:** Correlations of ICECAP-A and ICECAP-O scores and items with EQ-5D-5L, EQ VAS, happiness, satisfaction with life, WHO-5 and SWLS scores

Correlations^a^, age-group 18–64 years	Stability	Attachment	Autonomy	Achievement	Enjoyment	ICECAP-A
EQ-5D-5L index score	0.382	0.285	0.325	0.448	0.373	0.572
EQ VAS	0.382	0.292	0.3030	0.398	0.359	0.517
Happiness VAS (0–10)	0.299	0.322	0.225	0.316	0.380	0.501
Satisfaction with life VAS (0–10)	0.309	0.335	0.211	0.339	0.395	0.521
WHO-5 score	0.375	0.354	0.294	0.386	0.380	0.532
SWLS score	0.276	0.304	0.174	0.286	0.311	0.446

Correlations^a^, age-group 65 and over	Attachment	Security	Role	Enjoyment	Control	ICECAP-O
EQ-5D-5L score	0.347	0.494	0.584	0.511	0.506	0.649
EQ VAS	0.257	0.442	0.523	0.433	0.386	0.502
Happiness VAS (0–10)	0.364	0.411	0.448	0.428	0.341	0.516
Satisfaction with life VAS (0–10)	0.363	0.439	0.424	0.441	0.364	0.574
WHO-5 score	0.331	0.533	0.556	0.487	0.426	0.613
SWLS score	0.351	0.508	0.370	0.421	0.345	0.522

^a^Pearson correlations with ICECAP-A/-O scores, and Spearman’s rank correlation with ICECAP-A/-O items

For all correlations, *p* < 0.01

#### Correlations between the domains of ICECAP-A/-O and other measures (EQ-5D-5L, WHO-5, SWLS)

Correlation analysis between the ICECAP measures and EQ-5D-5L domains are provided in Online Resource 4. Correlation of the ICECAP-A score was the strongest with the Pain/discomfort EQ-5D-5L domain (*r* = − 0.534) and lowest with the Self-care domain (*r* = − 0.389). Correlation of the ICECAP-O score was the strongest with the Usual activities domain (*r* = − 0.572). Correlation between the ICECAP-A items and the EQ-5D-5L domains were moderate/weak, whilst between the ICECAP-O items and the EQ-5D-5L domains were weak to strong.

For both the ICECAP-A and ICECAP-O scores, the strongest correlations were observed with the WHO-5 statement “I have felt cheerful and in good spirits.” (*r* = 0.498 and 0.550) (Online Resource 5). Nevertheless, ICECAP-O score had strong correlation with all the WHO-5 statements, except for the one “I woke up feeling fresh and rested.” (*r* = 0.446). Correlation coefficient with ICECAP-A score was also the lowest for this statement (*r* = 0.377). Weak to moderate correlations were observed between the WHO-5 domains and the ICECAP-A items, whilst weak to strong correlations with the ICECAP-O items.

Regarding SWLS (Online Resource 6), both the ICECAP-A and the ICECAP-O had the strongest correlation with the statement “In most ways my life is close to my ideal.” (*r* = 0.441 and *r* = 0.540). Correlations between the SWLS domains and the ICECAP-A items were weak to moderate, and weak to strong with the ICECAP-O items.

#### Test–retest reliability

Among the 63 respondents aged < 65 years and 55 respondents aged 65+ who participated in the follow-up interview, 62 and 53 respondents answered all the 5 questions of the ICECAP-A and ICECAP-O questionnaires, respectively in both interviews. The average of ICECAP-A score was 0.88 (SD = 0.11) on the original interview and 0.89 (SD = 0.10) on the follow-up. Mean ICECAP-O scores were 0.86 (SD = 0.09) for both occasions. Test–retest ratings were fully consistent for all five items for 46 respondents (74.2%) for the ICECAP-A and for 44 participants (83.0%) for the ICECAP-O.

The ICC for the ICECAP-A instrument was 0.94 (95% confidence interval: 0.90–0.97), and 0.97 for the ICECAP-O instrument (95% confidence interval: 0.94–0.98), both indicating excellent agreement. On the ICECAP-A instrument, the agreement on item levels was the highest for Attachment (95.5%), lowest for Stability (85.5%), while it was 91.9% for Autonomy and Achievement and 93.5% for Enjoyment. Only 2 persons had larger than 1 level difference in ratings for Stability item and 1 person had larger than one level difference for Achievement. Regarding the ICECAP-O instrument, Attachment, Security and Control had an agreement of 96.2%, followed Enjoyment (94.3%), and Role (90.6%). Only 2 respondents had larger than 1 levels difference in the Role domain.

## Discussion

The aim of the paper was to develop the Hungarian language version of the ICECAP-A and ICECAP-O instruments and assess their psychometric properties in relation to various health status, well-being and social support measures. Moreover, we aimed to provide population norms with both instruments to be used as reference scores in further clinical and public health studies. Overall, the Hungarian ICECAP-A/-O versions showed good psychometric properties. Main socio-demographic characteristics were not significant determinants of the ICECAP-O and only a few associations were observed with the ICECAP-A.

In terms of the construct validity of the Hungarian ICECAP-A and ICECAP-O, associations were in line with intuitive assumptions and previous literature findings [9, 11, 12, 14]. Positive associations with marital status, employment, income, health and some major life events were confirmed by the subgroup analysis (and in multivariate regression analysis for the ICECAP-A), indicating good construct validity. Cronbach’s alpha of both ICECAP measures (0.86) indicated good internal consistency and these were very similar to what has been found by Linton et al. for the German version (0.83) and for the UK version (0.85) [11].

With respect to convergent validity, we assessed correlations between ICECAP measures and other standard validated health status and well-being measures such as EQ-5D-5L, WHO-5 and SWLS. Before we discuss the findings, we have to highlight that even though most of these questionnaires focus on some aspects of health and well-being, only the ICECAP measures build on the concept of capability, while all the others rather address functioning. This important conceptual difference can partly explain possible (and to some extent expected) divergence between ICECAP and the other measures. In addition, these measures apply to slightly different time frames that might also induce some disparity.

Correlations between ICECAP-A/-O scores and EQ-5D-5L index scores were strong, and also with EQ VAS. On the domains’ level, correlations between EQ-5D-5L domains and Autonomy (independence) of ICECAP-A were found weak, whilst were moderate in case of Control (independence) of ICECAP-O. We assume that younger (and healthier) individuals consider primarily other aspects than health when they think of autonomy (e.g. financial independence, autonomy in decision making).

Although most of the validity studies used the EQ-5D measures when assessing convergent validity of the ICECAP instruments [11, 14], only few of them are directly comparable to ours, as most of them used the three-response level (EQ-5D-3L) version [15, 18, 42], and focused on specific conditions or patient population rather than the general population. Correlation coefficient found in our study between the ICECAP-A score and EQ-5D-5L index score (*r* = 0.57), is very close to the ones found by Linton et al. for a German sample (*r* = 0.62) and for a UK sample (*r* = 0.61) of mixed populations of healthy individuals and patients [11]. In our study, the Pearson correlation coefficient between the ICECAP-O and the EQ-5D-5L score was 0.65. Hackert et al. found a very similar (Spearman) correlation coefficient of 0.63 on the sample of UK general population of elderly [24]. In both studies strongest correlations were observed between the Role item and the EQ-5D-5L domains, while lowest in the Attachment items and the EQ-5D-5L domains. Regarding ICECAP-O and EQ VAS scores, the corresponding coefficients were rather similar as well (0.58 vs 0.50).

With respect to the SWLS life-satisfaction measure, we found moderate/strong correlations between the ICECAP-A/O and SWLS scores, slightly lower than found in previous studies. Linton et al. reported stronger correlation between the SWLS and ICECAP-A scores (Germany: 0.66, the UK: 0.68) than between the EQ-5D-5L and ICECAP-A scores, indicating that ICECAP-A was more related to life satisfaction (SWLS) than to health-related quality of life. However, in our case, it was the opposite. Nevertheless, in both studies, correlation coefficients between SWLS and ICECAP-A domains were the lowest for the Autonomy domain. Regarding ICECAP-O, Hackert et al. reported a correlation coefficient of 0.72 between the ICECAP-O and SWLS scores [24], while in our case it was slightly lower, 0.52.

According to our knowledge, this is the first study to assess the relationship between the WHO-5 mental well-being measure and the ICECAP-A/-O. Given that both are well-being measures, although with different approach, we expected strong association between these tools. Correlations between ICECAP-A/O scores and WHO-5 score were indeed strong (0.53 and 0.61, respectively), nonetheless their strength remained slightly under the level seen between the ICECAP-A/-O and the EQ-5D-5L health status index.

To assess test–retest reliability of the instruments, about 5% of the respondents were asked to fill in the ICECAP-A and -O questionnaires right after the interviews. So far, only two studies explored test–retest reliability of the ICECAP-A [43, 44], and another two of the ICECAP-O instrument [42, 45]. In these studies, the follow-up interview took place 1–2 weeks after the baseline interview, thus we expected and in fact found higher intra-class coefficients in our study indicating excellent agreement.

Multiple regression analysis revealed no significant associations between socio-demographic characteristics and ICECAP-O. For the ICECAP-A, only income and having a paid job showed significant positive associations, whilst living in Budapest resulted significantly worse ICECAP-A scores. In Australia, Couzner et al. found no strong relationship between ICECAP-O and socio-demographic status in age-group 65 and over, either [16]. In our study, EQ-5D-5L health status was significant determinant of both ICECAP measures. Among the items of MEHM, only the self-perceived health status scale was proved to be significantly correlated with both ICECAP-A and -O (but the long-standing illness and GALI items were not). These results, on the one hand, enable estimates from one measure to another in the lack of observed data, nonetheless further research into exploring additional influencing factors of capabilities are suggested. On the other hand, our findings highlight and confirm previous studies regarding the remarkable difference between health status and well-being capabilities. We believe that it is time to consider the use of preference-based capability measures alongside the health status measures (e.g. MEHM) in international standardized and routinely collected population health statistics.

Some limitations of our study are worth mentioning. Because Hungarian tariffs for the ICECAP-A/-O and EQ-5D-5L were not available at the time of the study, the tariffs for the UK and England were used, respectively. These might differ from the preferences of the Hungarian general population. Also, the English tariff for the EQ-5D-5L has been recently criticized in the literature [46]. Therefore, we analysed the correlations between these measures on the domains’ level as well which are independent from the national tariffs. Due to the cross-sectional nature of the study, we could not assess the responsiveness of the Hungarian ICECAP instruments to changes. Further prospective studies should be undertaken to tackle this issue. The time between the test and retest was short, therefore we cannot exclude some recall effects. This validation study has been carried out on a representative sample of the general population, which we think is one of the strengths of our research. Nevertheless, validity of the instruments should be tested also on patient populations for use in disease-specific studies.

## Conclusion

To the best of our knowledge, this is the first study in Europe to present population norms with the ICECAP-A and ICECAP-O measures based on a large representative sample. Being the first study in the Central and Eastern European region, our study contributes to the growing literature exploring the psychometric validity of ICECAP measures across different cultural contexts. Our results proved that the Hungarian language versions of the ICECAP-A and ICECAP-O instruments are valid and reliable measures of capability among the general population. Our results provide a basis for estimating ICECAP-A/-O inputs for health economic analyses from regularly collected population health statistics (self-perceived health status scale and WHO-5) in the lack of observed data, although we encourage widespread direct measurements. Health is an important determinant of well-being capabilities, but main socio-demographic characteristics have no or only minor impact on ICECAP-O and ICECAP-A, respectively. Other influencing factors deserve further investigation in future research.

## Electronic supplementary material

Below is the link to the electronic supplementary material.

Supplementary file1 (DOCX 53 kb)

## Data Availability

The data that support the findings of this study are available from the corresponding author upon reasonable request.
