# Supplementary material for: Capability of well-being: validation of the Hungarian version of the ICECAP-A and ICECAP-O questionnaires and population normative data
Source: Qual Life Res. 2020 May 28;29(10):2863–74. doi: 10.1007/s11136-020-02542-1 (PMC7561558; doi:10.1007/s11136-020-02542-1)
Supplement: Supplementary file 1 — Supplementary file1 (DOCX 53 kb) [file 11136_2020_2542_MOESM1_ESM.docx]

Title: Capability of well-being: Validation of the Hungarian version of the ICECAP-A and ICECAP-O questionnaires and population normative data

Authors: Petra Baji, Miklós Farkas, Ágota Dobos, Zsombor Zrubka, László Gulácsi, Valentin Brodszky, Fanni Rencz, Márta Péntek

Journal: Quality of Life Research

Corresponding author:

Márta Péntek, Department of Health Economics, Corvinus University of Budapest; E-mail address: [marta.pentek@uni-corvinus.hu](mailto:marta.pentek@uni-corvinus.hu)

**Online Resource 1: ICECAP-A, ICECAP-O distribution of responses**

| ICECAP-A (age <65), N=1568 | N | % | ICECAP-O (age 65+), N=453 | N | % |
| --- | --- | --- | --- | --- | --- |
| **Feeling settled and secure (Stability)** |  |  | **Love and Friendship (Attachment)** |  |  |
| 4 I am able to feel settled and secure in all areas of my life | 813 | 51.8% | 4 I can have all of the love and friendship that I want | 159 | 35.1% |
| 3 I am able to feel settled and secure in many areas of my life | 602 | 38.4% | 3 I can have a lot of the love and friendship that I want | 238 | 52.5% |
| 2 I am able to feel settled and secure in a few areas of my life | 132 | 8.4% | 2 I can have a little of the love and friendship that I want | 53 | 11.7% |
| 1 I am unable to feel settled and secure in any areas of my life | 21 | 1.3% | 1 I cannot have any of the love and friendship that I want | 3 | 0.7% |
| **Love, friendship and support (Attachment)** |  |  | **Thinking about the future (Security)** |  |  |
| 4 I can have a lot of love, friendship and support | 932 | 59.4% | 4 I can think about the future without any concern | 110 | 24.3% |
| 3 I can have quite a lot of love, friendship and support | 564 | 36.0% | 3 I can think about the future with only a little concern | 208 | 45.9% |
| 2 I can have a little love, friendship and support | 69 | 4.4% | 2 I can only think about the future with some concern | 94 | 20.8% |
| 1 I cannot have any love, friendship and support | 3 | 0.2% | 1 I can only think about the future with a lot of concern | 41 | 9.1% |
| **Being independent (Autonomy)** |  |  | **Doing things that make you feel valued (Role)** |  |  |
| 4 I am able to be completely independent | 825 | 52.6% | 4 I am able to do all of the things that make me feel valued | 123 | 27.2% |
| 3 I am able to be independent in many things | 616 | 39.3% | 3 I am able to do many of the things that make me feel valued | 210 | 46.4% |
| 2 I am able to be independent in a few things | 115 | 7.3% | 2 I am able to do a few of the things that make me feel valued | 111 | 24.5% |
| 1 I am unable to be at all independent | 12 | 0.8% | 1 I am unable to do any of the things that make me feel valued | 9 | 2.0% |
| **Achievement and progress (Achievement)** |  |  | **Enjoyment and pleasure (Enjoyment)** |  |  |
| 4 I can achieve and progress in all aspects of my life | 681 | 43.4% | 4 I can have all of the enjoyment and pleasure that I want | 98 | 21.6% |
| 3 I can achieve and progress in many aspects of my life | 699 | 44.6% | 3 I can have a lot of the enjoyment and pleasure that I want | 265 | 58.5% |
| 2 I can achieve and progress in a few aspects of my life | 172 | 11.0% | 2 I can have a little of the enjoyment and pleasure that I want | 85 | 18.8% |
| 1 I cannot achieve and progress in any aspects of my life | 16 | 1.0% | I cannot have any of the enjoyment and pleasure that I want | 5 | 1.1% |
| **Enjoyment and pleasure (Enjoyment)** |  |  | **Independence (Control)** |  |  |
| 4 I can have a lot of enjoyment and pleasure | 887 | 56.6% | 4 I am able to be completely independent | 131 | 28.9% |
| 3 I can have quite a lot of enjoyment and pleasure | 581 | 37.1% | 3 I am able to be independent in many things | 220 | 48.6% |
| 2 I can have a little enjoyment and pleasure | 95 | 6.1% | 2 I am able to be independent in a few things | 89 | 19.6% |
| 1 I cannot have any enjoyment and pleasure | 5 | 0.3% | 1 I am unable to be at all independent | 13 | 2.9% |

**Online Resource 2: Results of the OLS regression analysis**

|  | (1) | (2) | (3) | (4) |
| --- | --- | --- | --- | --- |
| VARIABLES | ICECAP-A score | ICECAP-A score | ICECAP-O score | ICECAP-O score |
|  |  |  |  |  |
| Woman | -0.000607 | -0.000852 | 0.0145 | 0.0152 |
| (Base: man) | (-0.0957) | (-0.133) | (0.945) | (0.953) |
| Age | 6.35e-05 | 0.000116 | 0.00160 | 0.00139 |
|  | (0.225) | (0.408) | (1.345) | (1.211) |
| Educ: primary | -0.00563 | -0.00575 | 0.00862 | 0.0131 |
| (Base: tertiary) | (-0.697) | (-0.711) | (0.317) | (0.450) |
| Educ: secondary | -0.00216 | -0.00177 | 0.0206 | 0.0217 |
| (Base: tertiary) | (-0.301) | (-0.247) | (0.848) | (0.843) |
| Residence: capital | -0.0237*** | -0.0241*** | -0.0158 | -0.0167 |
| (Base: town) | (-3.221) | (-3.280) | (-0.897) | (-0.958) |
| Residence: village | 0.00254 | 0.00184 | 0.0109 | 0.00595 |
| (Base: town) | (0.344) | (0.249) | (0.730) | (0.417) |
| Married/partner: yes | 0.0112 | 0.0120* | 0.0218 | 0.0233 |
| (Base no) | (1.618) | (1.728) | (1.402) | (1.526) |
| Paid job: yes | 0.0208** | 0.0201** | 0.0248 | 0.0282 |
| (Base: no) | (2.200) | (2.098) | (1.128) | (1.336) |
| Per capita net monthly income | 0.000237*** | 0.000239*** | 0.000323 | 0.000322 |
|  | (3.433) | (3.460) | (1.475) | (1.480) |
| Household size | 0.00599* | 0.00622* | 0.00421 | 0.00337 |
|  | (1.731) | (1.774) | (0.314) | (0.254) |
| EQ-5D-5L score | 0.379*** | 0.365*** | 0.336*** | 0.351*** |
|  | (6.660) | (6.531) | (6.059) | (5.760) |
| EQ VAS | 0.00136*** | 0.00141*** | 0.00119** | 0.00129** |
|  | (4.118) | (4.142) | (2.193) | (2.379) |
| Self-perceived health: very poor | -0.0179 | -0.0176 | -0.0912 | -0.0961 |
| Base: very good | (-0.415) | (-0.436) | (-1.171) | (-1.370) |
| Self-perceived health: poor | -0.153*** | -0.140*** | -0.0931** | -0.0983** |
| Base: very good | (-4.426) | (-4.381) | (-2.205) | (-2.366) |
| Self-perceived health: fair | -0.0527*** | -0.0554*** | -0.0346 | -0.0399 |
| Base: very good | (-4.303) | (-4.800) | (-1.248) | (-1.404) |
| Self-perceived health: good | -0.0271*** | -0.0271*** | -0.0520* | -0.0472 |
| Base: very good | (-4.299) | (-4.318) | (-1.752) | (-1.566) |
| Long-standing illness: yes | 0.00273 | - | 5.16e-06 | - |
| (base: no) | (0.271) | - | (0.000355) | - |
| GALI: severely limited | -0.0580 | - | -0.00221 | - |
| (base: not limited) | (-1.348) | - | (-0.0460) | - |
| GALI: limited but not severely | -0.0506 | - | 0.0227 | - |
| (base: not limited) | (-1.150) | - | (0.426) | - |
| Constant | 0.429*** | 0.386*** | 0.321* | 0.330* |
|  | (6.703) | (7.753) | (1.841) | (1.944) |
|  |  |  |  |  |
| Observations | 1028 | 1030 | 308 | 312 |
| R-squared | 0.444 | 0.443 | 0.505 | 0.498 |
| F > test | 27.86 | 32.23 | 12.10 | 13.06 |
| p | 0.0000 | 0.0000 | 0.0000 | 0.0000 |

Robust t-statistics in parentheses

*** p<0.01, ** p<0.05, * p<0.1

**Online Resource 3: ICECAP-A and ICECAP-O scores by life events in the last 12 months with ANOVA test**

| **Life events** | **N** | **%** | **ICECAP-A**  **Mean (SD)** | **N** | **%** | **ICECAP-O**  **Mean (SD)** |
| --- | --- | --- | --- | --- | --- | --- |
| Death of a close relative or friend |  |  | **F=7.89**  **p=0.0050** |  |  | **F=8.13**  **p=0.0046** |
| Yes | 167 | 10.7% | 0.87 (0.14) | 91 | 20.1% | 0.79 (0.16) |
| No | 1401 | 89.3% | 0.9 (0.12) | 362 | 79.9% | 0.84 (0.15) |
| Divorce, separation or break-up of an intimate relationship |  |  | **F=0.72**  **P=0.3959** |  |  | **F=2.66**  **P=0.1039** |
| Yes | 57 | 3.6% | 0.88 (0.16) | 6 | 1.3% | 0.73 (0.26) |
| No | 1511 | 96.4% | 0.89 (0.12) | 447 | 98.7% | 0.83 (0.15) |
| A wage earner in your household losing their job |  |  | **F=10.27**  **P=0.0014** |  |  | **F=0.71**  **P=0.3997** |
| Yes | 51 | 3.3% | 0.84 (0.16) | 2 | 0.4% | 0.92 (0.01) |
| No | 1517 | 96.7% | 0.9 (0.12) | 451 | 99.6% | 0.83 (0.15) |
| Changing your job |  |  | **F=0.71**  **P=0.4008** |  |  | **F=0.03**  **P=0.8737** |
| Yes | 142 | 9.1% | 0.89 (0.14) | 1 | 0.2% | 0.85 (0) |
| No | 1426 | 90.9% | 0.9 (0.12) | 452 | 99.8% | 0.83 (0.15) |
| Having serious financial worries or debts |  |  | **F=188.74**  **P=0.0000** |  |  | **F=14.37**  **P=0.0002** |
| Yes | 87 | 5.5% | 0.72 (0.19) | 2 | 0.5% | 0.71 (0.17) |
| No | 1481 | 94.5% | 0.9 (0.11) | 433 | 99.5% | 0.83 (0.15) |
| Moving house |  |  | **F=0.25**  **P=0.6137** |  |  | **F=0.06**  **P=0.8025** |
| Yes | 134 | 8.5% | 0.89 (0.12) | 10 | 2.2% | 0.82 (0.23) |
| No | 1434 | 91.5% | 0.89 (0.13) | 443 | 97.8% | 0.83 (0.15) |
| Problems with parents or close relatives |  |  | **F=30.00**  **P=0.0000** |  |  | **F=4.81**  **P=0.0288** |
| Yes | 102 | 6.5% | 0.83 (0.16) | 11 | 2.4% | 0.73 (0.23) |
| No | 1466 | 93.5% | 0.9 (0.12) | 442 | 97.6% | 0.83 (0.15) |
| Problems with your children |  |  | **F=0.57**  **P=0.4520** |  |  | **F=5.65**  **P=0.0179** |
| Yes | 79 | 5.0% | 0.88 (0.14) | 24 | 5.3% | 0.76 (0.19) |
| No | 1489 | 95.0% | 0.89 (0.13) | 429 | 94.7% | 0.83 (0.15) |
| Problems at work |  |  | **F=5.87**  **P=0.0156** |  |  | **F=0.07**  **P=0.7884** |
| Yes | 135 | 8.6% | 0.87 (0.13) | 1 | 0.2% | 0.87 (0) |
| No | 1433 | 91.4% | 0.9 (0.13) | 452 | 99.8% | 0.83 (0.15) |
| Problems with neighbours |  |  | **F=0.45**  **P=0.5030** |  |  | **F=3.17**  **P=0.0758** |
| Yes | 93 | 5.9% | 0.89 (0.13) | 35 | 7.7% | 0.78 (0.19) |
| No | 1475 | 94.1% | 0.89 (0.13) | 418 | 92.3% | 0.83 (0.15) |
| A serious accident or injury to you |  |  | **F=25.91**  **P=0.0000** |  |  | **F=1.93**  **P=0.1650** |
| Yes | 31 | 2.0% | 0.78 (0.21) | 11 | 2.4% | 0.77 (0.17) |
| No | 1537 | 98.0% | 0.9 (0.12) | 442 | 97.6% | 0.83 (0.15) |
| Other serious illness to you |  |  | **F=202.61**  **P=0.0000** |  |  | **F=55.66**  **P=0.0000** |
| Yes | 58 | 3.7% | 0.68 (0.2) | 66 | 14.6% | 0.71 (0.22) |
| No | 1510 | 96.3% | 0.9 (0.12) | 387 | 85.4% | 0.85 (0.12) |
| None of these |  |  | **F=82.66**  **P=0.0000** |  |  | **F=40.75**  **P=0.0000** |
| Yes | 907 | 57.8% | 0.92 (0.1) | 258 | 57.0% | 0.87 (0.11) |
| No | 661 | 42.2% | 0.86 (0.15) | 195 | 43.0% | 0.78 (0.18) |

**Online Resource 4: Correlations* of ICECAP-A and ICECAP-O items with the EQ-5D-5L domains**

| **Correlations*,**  **age-group 18-64 years** | **Stability** | **Attachment** | **Autonomy** | **Achievement** | **Enjoyment** | **ICECAP-A** |
| --- | --- | --- | --- | --- | --- | --- |
| Mobility | -0.305 | -0.223 | -0.254 | -0.336 | -0.292 | -0.471 |
| Self-care | -0.229 | -0.180 | -0.196 | -0.236 | -0.169 | -0.389 |
| Usual activities | -0.282 | -0.218 | -0.242 | -0.347 | -0.269 | -0.447 |
| Pain/discomfort | -0.370 | -0.259 | -0.298 | -0.412 | -0.330 | -0.534 |
| Anxiety/depression | -0.280 | -0.267 | -0.244 | -0.325 | -0.324 | -0.429 |
| **Correlations*,**  **age-group 65 years and over** | **Attachment** | **Security** | **Role** | **Enjoyment** | **Control** | **ICECAP-O** |
| Mobility | -0.221 | -0.350 | -0.506 | -0.395 | -0.417 | -0.498 |
| Self-care | -0.264 | -0.360 | -0.466 | -0.375 | -0.422 | -0.538 |
| Usual activities | -0.287 | -0.442 | -0.565 | -0.480 | -0.444 | -0.572 |
| Pain/discomfort | -0.290 | -0.437 | -0.498 | -0.455 | -0.411 | -0.526 |
| Anxiety/depression | -0.304 | -0.427 | -0.352 | -0.371 | -0.362 | -0.518 |

*Pearson correlations with ICECAP-A/-O scores, and Spearman’s rank correlation with ICECAP-A/-O items.

For all correlations p<0.01.

**Online Resource 5: Correlations* of ICECAP-A and ICECAP-O items with the WHO-5 domains**

| **Correlations*, age-group 18-64 years** | **Stability** | **Attachment** | **Autonomy** | **Achievement** | **Enjoyment** | **ICECAP-A** |
| --- | --- | --- | --- | --- | --- | --- |
| I have felt cheerful and in good spirits. | 0.316 | 0.356 | 0.261 | 0.344 | 0.357 | 0.498 |
| I have felt calm and relaxed. | 0.310 | 0.302 | 0.253 | 0.313 | 0.331 | 0.426 |
| I have felt active and vigorous. | 0.301 | 0.269 | 0.228 | 0.311 | 0.296 | 0.436 |
| I woke up feeling fresh and rested. | 0.303 | 0.246 | 0.231 | 0.317 | 0.261 | 0.377 |
| My daily life has been filled with things that interest me. | 0.284 | 0.284 | 0.222 | 0.294 | 0.321 | 0.432 |
| **Correlations*, age-group 65 years and over** | **Attachment** | **Security** | **Role** | **Enjoyment** | **Control** | **ICECAP-O** |
| I have felt cheerful and in good spirits. | 0.295 | 0.483 | 0.488 | 0.445 | 0.395 | 0.550 |
| I have felt calm and relaxed. | 0.303 | 0.477 | 0.399 | 0.401 | 0.356 | 0.531 |
| I have felt active and vigorous. | 0.254 | 0.394 | 0.520 | 0.360 | 0.377 | 0.513 |
| I woke up feeling fresh and rested. | 0.215 | 0.425 | 0.440 | 0.381 | 0.328 | 0.446 |
| My daily life has been filled with things that interest me. | 0.338 | 0.451 | 0.471 | 0.459 | 0.372 | 0.523 |

* Pearson correlations with ICECAP-A/-O scores, and Spearman’s rank correlation with ICECAP-A/-O items.

For all correlations p<0.01.

**Online Resource 6: Correlations* of ICECAP-A and ICECAP-O items with the Satisfaction with Life Scale (SWLS) domains**

| **Correlations*, age-group 18-64 years** | **Stability** | **Attachment** | **Autonomy** | **Achievement** | **Enjoyment** | **ICECAP-A** |
| --- | --- | --- | --- | --- | --- | --- |
| In most ways my life is close to my ideal. | 0.263 | 0.298 | 0.199 | 0.283 | 0.326 | 0.441 |
| The conditions of my life are excellent. | 0.220 | 0.284 | 0.157 | 0.236 | 0.288 | 0.374 |
| I am satisfied with my life. | 0.232 | 0.293 | 0.146 | 0.223 | 0.303 | 0.407 |
| So far I have gotten the important things I want in life. | 0.189 | 0.167 | 0.122 | 0.188 | 0.182 | 0.279 |
| If I could live my life over, I would change almost nothing. | 0.218 | 0.227 | 0.108 | 0.233 | 0.215 | 0.318 |
| **Correlations*, age-group 65 years and over** | **Attachment** | **Security** | **Role** | **Enjoyment** | **Control** | **ICECAP-O** |
| In most ways my life is close to my ideal. | 0.328 | 0.507 | 0.444 | 0.436 | 0.373 | 0.540 |
| The conditions of my life are excellent. | 0.324 | 0.452 | 0.351 | 0.385 | 0.342 | 0.450 |
| I am satisfied with my life. | 0.308 | 0.407 | 0.309 | 0.345 | 0.337 | 0.471 |
| So far I have gotten the important things I want in life. | 0.215 | 0.312 | 0.181 | 0.246 | 0.232 | 0.337 |
| If I could live my life over, I would change almost nothing. | 0.226 | 0.314 | 0.165 | 0.249 | 0.091 | 0.271 |

* Pearson correlations with ICECAP-A/-O scores, and Spearman’s rank correlation with ICECAP-A/-O items.

For all correlations p<0.01, except between ‘Control’ item (ICECAP-O) and ’If I could live my life over, I would change almost nothing.’ domain of SWLS.
